# Supplementary material for: Specific Learning Disabilities and Emotional-Behavioral Difficulties: Phenotypes and Role of the Cognitive Profile
Source: J Clin Med. 2023 Feb 27;12(5):1882. doi: 10.3390/jcm12051882 (PMC10003319; doi:10.3390/jcm12051882)
Supplement: Supplementary file 1 [file jcm-12-01882-s001.zip › jcm-2208713-supplementary.pdf]

APPENDIX

Table S1. *t* statistics for Bonferroni post-hoc corrections for multiple comparisons, on the Wechsler indices

WISC-IV indices	*t*	*df*	*p*
VCI - PRI	-1,930	116	0,596
PRI - WMI	10,958	114	0,000*
WMI - PSI	-3,927	114	0,000*
PSI - VCI	-4,864	113	0,000*
VCI - WMI	10,191	113	0,000*
PSI - PRI	-6,493	114	0,000*

. * represent the statistical significant differences (p<.05) across Wechsler indices or CBCL scales.

Table S2. *t* statistics for Bonferroni post-hoc corrections for multiple comparisons on the CBCL DSM-oriented sub-scales

CBCL DSM-oriented sub-scales	*t*	*df*	*p*
Affective Problem - Anxiety Problem	-3,015	120	0,047*
Affective Problem - Somatic Problem	3,693	120	0,005*
Affective Problem - Attention Deficit/Hyperactivity Problem	1,711	120	1,000
Affective Problem - Oppositional Defiant Problems	5,859	120	0,000*
Affective Problem - Conduct Problems	7,613	120	0,000*
Anxiety Problem - Somatic Problem	5,680	120	0,000*
Anxiety Problem - Attention Deficit/Hyperactivity Problem	4,078	120	0,001*
Anxiety Problem - Oppositional Defiant Problems	8,049	120	0,000*
Anxiety Problem - Conduct Problems	9,395	120	0,000*
Somatic Problem - Attention Deficit/Hyperactivity Problem	-2,009	120	0,701
Somatic Problem - Oppositional Defiant Problems	0,941	120	1,000
Somatic Problem - Conduct Problems	2,943	120	0,058*
Attention Deficit/Hyperactivity Problem - Oppositional Defiant Problems	4,506	120	0,000*
Attention Deficit/Hyperactivity Problem - Conduct Problems	7,079	120	0,000*
Oppositional Defiant Problems - Conduct Problems	3,639	120	0,060

. * represent the statistical significant differences (p<.05) across Wechsler indices or CBCL scales.

Table S3. *t* statistics for Bonferroni post-hoc corrections for multiple comparisons on the CBCL syndrome scales

**CBCL syndrome subscales**	***t***	***df***	***p***
Anxious/Depressed - With-drawn/Depressed	1,101	120	1,000
Anxious/Depressed - Somatic Complaints	3,230	120	0,045*
Anxious/Depressed - Social Problems	1,940	120	1,000
Anxious/Depressed - Thought Problems	6,013	120	0,000*
Anxious/Depressed - Attention Problem	-1,297	120	1,000
Anxious/Depressed - Rule Breaking Behaviour	6,915	120	0,000*
Anxious/Depressed - Aggressive Behaviour	5,939	120	0,000*
With-drawn/Depressed - Somatic Complaints	2,187	120	0,859
With-drawn/Depressed - Social Problems	0,741	120	1,000
With-drawn/Depressed - Thought Problems	4,090	120	0,002*
With-drawn/Depressed - Attention Problem	-2,034	120	1,000
With-drawn/Depressed - Rule Breaking Behaviour	6,255	120	0,000*
With-drawn/Depressed - Aggressive Behaviour	4,559	120	0,000*
Somatic Complaints - Social Problems	-1,598	120	1,000
Somatic Complaints - Thought Problems	2,232	120	0,769
Somatic Complaints - Attention Problem	-3,918	120	0,004*
Somatic Complaints - Rule Breaking Behaviour	4,163	120	0,002*
Somatic Complaints - Aggressive Behaviour	2,501	120	0,384
Social Problems - Thought Problems	3,948	120	0,004*
Social Problems - Attention Problem	-3,148	120	0,580
Social Problems - Rule Breaking Behaviour	6,609	120	0,000*
Social Problems - Aggressive Behaviour	4,616	120	0,000*
Thought Problems - Attention Problem	-6,006	120	0,000*
Thought Problems - Rule Breaking Behaviour	2,356	120	0,563
Thought Problems - Aggressive Behaviour	0,362	120	1,000
Attention Problem - Rule Breaking Behaviour	8,847	120	0,000*
Attention Problem - Aggressive Behaviour	7,312	120	0,000*
Rule Breaking Behaviour - Aggressive Behaviour	-2,814	120	0,160

. * represent the statistical significant differences (p<.05) across Wechsler indices or CBCL scales.
